# ‘Resistance is futile?’ – paradoxical inhibitory effects of K_ATP_ channel closure in glucagon‐secreting α‐cells

**DOI:** 10.1113/JP279775

**Published:** 2020-08-07

**Authors:** Quan Zhang, Haiqiang Dou, Patrik Rorsman

**Affiliations:** ^1^ Oxford Centre for Diabetes Endocrinology and Metabolism Radcliffe Department of Medicine University of Oxford Churchill Hospital Oxford OX3 7LE UK; ^2^ Metabolic Physiology Unit Institute of Neuroscience and Physiology University of Göteborg PO Box 430 Göteborg SE‐405 30 Sweden

**Keywords:** diabetes, glucagon, insulin, K_ATP_ channels, membrane potential

## Abstract

By secreting insulin and glucagon, the β‐ and α‐cells of the pancreatic islets play a central role in the regulation of systemic metabolism. Both cells are equipped with ATP‐regulated potassium (K_ATP_) channels that are regulated by the intracellular ATP/ADP ratio. In β‐cells, K_ATP_ channels are active at low (non‐insulin‐releasing) glucose concentrations. An increase in glucose leads to K_ATP_ channel closure, membrane depolarization and electrical activity that culminates in elevation of [Ca^2+^]_i_ and initiation of exocytosis of the insulin‐containing secretory granules. The α‐cells are also equipped with K_ATP_ channels but they are under strong tonic inhibition at low glucose, explaining why α‐cells are electrically active under hypoglycaemic conditions and generate large Na^+^‐ and Ca^2+^‐dependent action potentials. Closure of residual K_ATP_ channel activity leads to membrane depolarization and an increase in action potential firing but this stimulation of electrical activity is associated with inhibition rather than acceleration of glucagon secretion. This paradox arises because membrane depolarization reduces the amplitude of the action potentials by voltage‐dependent inactivation of the Na^+^ channels involved in action potential generation. Exocytosis in α‐cells is tightly linked to the opening of voltage‐gated P/Q‐type Ca^2+^ channels, the activation of which is steeply voltage‐dependent. Accordingly, the inhibitory effect of the reduced action potential amplitude exceeds the stimulatory effect resulting from the increased action potential frequency. These observations highlight a previously unrecognised role of the action potential amplitude as a key regulator of pancreatic islet hormone secretion.

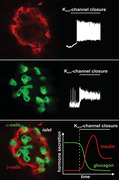

## Graphical abstract

**Abstract figure legend** The confocal images of a mouse pancreatic islet with β‐cells in red (top left), α‐cells in green (middle left) and merged image (bottom left). The images were taken at the top of an islet using an upright confocal microscope, which is why a clear separation of the β‐ and α‐cells is not as evident as in the centre of the islet. Panels to the right show the effects of K_ATP_ channel closure on electrical activity in β‐ (top right) and α‐cells (middle right). The schematic (lower right) summarizes the relationship between K_ATP_ channel closure/electrical activity on insulin and glucagon secretion. A decrease in K_ATP_ channel activity leads to a biphasic stimulation of insulin secretion (from a low basal value) but inhibition of glucagon secretion (from a high basal rate) despite similar effects on electrical activity in α‐ and β‐cells. This Topical Review discusses this paradox.

## Introduction

In most cells, potassium (K^+^) channel activity constitutes the principal background membrane conductance, which explains why the resting membrane potential usually approximates the K^+^ equilibrium potential (E_K_; ∼‐70 mV). In general, an increase in K^+^ channel activity lowers electrical excitability by clamping the membrane potential at E_K_ and reducing the input resistance; both factors making it more difficult for the cell to generate action potentials. Conversely, a decrease in K^+^ channel activity increases electrical excitability by producing membrane depolarization and increasing the membrane resistance. Under the latter conditions, the opening of individual Na^+^ or Ca^2+^ channels may suffice for the cell to depolarize to the threshold for action potential firing (Fenwick *et al*. 1982).

ATP‐sensitive potassium (K_ATP_) channels are expressed in a number of cells including cardiomyocytes, skeletal muscle, smooth muscle cells and certain neurones (Huang *et al*. 2019). However, their role is particularly evident in the β‐cells of the pancreatic islets (Ashcroft, 2007). The pancreatic islets represent the endocrine part of the pancreas and make up about 1% of its mass, corresponding to ∼1 g in humans (Rorsman & Ashcroft, 2018). The islets are micro‐organs consisting of (on average) ∼200 endocrine cells: insulin‐producing β‐cells (75% of islet cells) and glucagon‐producing α‐cells (15–20%). In addition, there are a small number (5%) of somatostatin‐releasing δ‐cells. Insulin and glucagon are the body's principal glucose‐regulating hormones (Frayn & Evans, 2019). They are released in response to increases (hyperglycaemia) and decreases (hypoglycaemia) in plasma glucose concentrations, respectively. Collectively they ensure that plasma glucose is maintained at ∼5 mm in humans.

In β‐cells, an increase in plasma glucose (for example, following ingestion of a carbohydrate‐rich meal) stimulates insulin secretion by closing K_ATP_ channels (Rorsman & Ashcroft, 2018). Intriguingly, K_ATP_ channels are also found in the glucagon‐secreting α‐cells (Bokvist *et al*. 1999). Given the reciprocal regulation of insulin and glucagon secretion by glucose, the question arises as to how K_ATP_ channel closure *stimulates* secretion in β‐cells but *inhibits* it in α‐cells. In this Topical Review we consider this conundrum.

## K_ATP_ channels

K_ATP_ channels are octameric heterocomplexes of four KIR6.2 (encoded by *Kcnj11*) and four SUR1 (encoded by *Abcc8*) subunits (Seino, 1999). They are regulated by variations of the intracellular ATP concentrations: they are active in the absence of ATP and are inhibited by increasing concentrations of ATP with an IC_50_ of ∼10μm in both α‐ and β‐cells (Cook & Hales, 1984; Bokvist *et al*. 1999). K_ATP_ channels closed by ATP can be reactivated by Mg‐ADP (Dunne & Petersen, 1986; Kakei *et al*. 1986) and in the intact cell the channel activity is therefore believed to be regulated by the submembrane ATP/ADP ratio.

The K_ATP_ channel is the molecular target of the hypoglycaemic sulphonylureas (SUs) (Trube *et al*. 1986), compounds that have been used to treat type‐2 diabetes (T2D) since the early 1950s. Tolbutamide is an example of the first generation SUs and blocks the K_ATP_ channels with an IC_50_ of 7 μm; second generation SUs (like glibenclamide) inhibit channel activity at nanomolar concentrations. Conversely, the K_ATP_ channels are activated by K^+^ channel openers such as diazoxide, which activates the channel with an EC_50_ of 20–30 μm in β‐ and α‐cells (Zunkler *et al*. 1988; Zhang *et al*. 2013).

Because of its central role in the control of systemic metabolism, the β‐cell can be regarded as the archetypal metabolically regulated cell. We will therefore start by summarizing the role of the K_ATP_ channel in insulin secretion. We will then consider the more controversial role of K_ATP_ channels in the glucagon‐secreting α‐cell against the backdrop of the β‐cell. There are several excellent reviews on the structure and function of the K_ATP_ channels and the respective roles of the two subunits in its regulation (Seino, 1999; Nichols, 2006; McTaggart *et al*. 2010). These aspects will therefore not be reviewed here.

## K_ATP_ channels in metabolic sensing: the β‐cell

**Figure 1:**
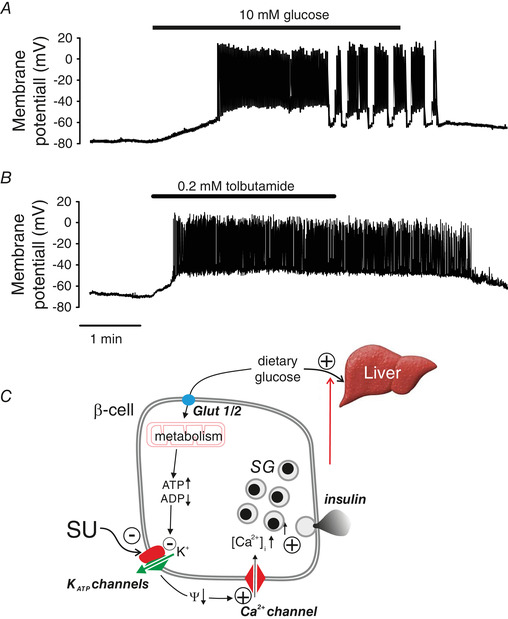
K_ATP_ channels in β‐cells *A*, electrical activity in β‐cells evoked by an increase in plasma glucose from 1 to 10 mm. *B*, as in *A* but tolbutamide (0.2 mm) applied instead of glucose. *C*, schematic of consensus model of glucose‐induced insulin secretion in β‐cells. Glut1/2, glucose transporters 1 and 2 (human and mouse, respectively); K_ATP_ channels, ATP‐sensitive K^+^ channels; Ψ, membrane potential; SG, secretory granules. The + and ‐ signs denote stimulation and inhibition, respectively, whereas the arrows (↑,↓) indicate an increase or decrease of the indicated parameter. The red arrow indicates feedback regulation of insulin secretion via changes in plasma glucose due to (for example) hepatic glucose disposition.

β‐cell electrical activity is tightly correlated with insulin secretion (Rorsman & Ashcroft, 2018). At low (1 mm) glucose (when insulin secretion is suppressed), the β‐cell is hyperpolarised (‐70 mV) and electrically silent. Increasing glucose to concentrations ≥6 mm (i.e. close to the normal plasma glucose levels) induces membrane depolarization. When the membrane potential reaches a threshold of ∼‐50 mV, the β‐cells start generating oscillatory electrical activity consisting of bursts of action potentials that originate from depolarized plateaux separated by electrically silent repolarized intervals. The bursts of action potentials leads to increases in [Ca^2+^]_i_ that trigger exocytosis of the insulin‐containing secretory granules by mechanisms similar to those involved in neurotransmitter release (Fig. 1*A* and *C*) (Rorsman & Ashcroft, 2018). As in neurones, exocytosis of the insulin‐containing granules appears to be determined by the local [Ca^2+^]_i_ in close proximity to the inner mouth of the Ca^2+^ channels (Pertusa *et al*. 1999; Barg *et al*. 2001).

In β‐cells, the glucose‐induced membrane depolarization and associated initiation of action potential firing are mediated by changes in K_ATP_ channel activity. Under hypoglycaemic conditions, K_ATP_ channel activity is high because of a low cytoplasmic ATP/ADP ratio and the combination of a negative membrane potential and low membrane resistance prevents electrical activity. When plasma glucose rises, accelerated glucose uptake into the β‐cells and the associated stimulation of metabolism increase the cytoplasmic ATP/ADP ratio and thereby causes K_ATP_ channel closure.

When K_ATP_ channel activity is reduced, the β‐cells depolarize because the residual K^+^ permeability is no longer sufficient to counteract the depolarizing influence of other membrane conductances that were previously too small to affect the membrane potential. The identity of the depolarizing background membrane conductance remains to be unequivocally identified; candidates include TRPM2 or Piezo1 (Yosida *et al*. 2014; Deivasikamani *et al*. 2019) and/or Cl^−^‐permeable LRRC8 channels (also known as SWELL1 or VRAC) (Stuhlmann *et al*. 2018).

As reviewed previously (Rorsman & Ashcroft, 2018), β‐cell electrical activity reflects the activation of voltage‐gated Ca^2+^ and K^+^ channels, which underlie the up‐ and downstroke of the action potential, respectively. In mouse β‐cells, the Ca^2+^ channels are L‐ and R‐type whereas in human β‐cells, L‐ and P/Q‐type Ca^2+^ channels predominate. Voltage‐gated Na^+^ channels also contribute but mainly at glucose concentrations just above the threshold for insulin secretion (Braun *et al*. 2008). However, in mouse β‐cells the Na^+^ channels exhibit an unusual voltage dependence of inactivation and in most cells they are completely inactivated at the normal resting potential of ‐70 mV (Zhang *et al*. 2014; Godazgar *et al*. 2018).

The repolarization of the action potential results from the opening of Kv2.1 (mouse) or Kv2.2 (human) and large‐conductance Ca^2+^‐activated K^+^ channels (Jacobson *et al*. 2007; Braun *et al*. 2008; Houamed *et al*. 2010). Pharmacological inhibition of the voltage‐gated K^+^ channels amplifies glucose‐induced insulin secretion by increasing the duration and amplitude of the action potentials (Rorsman *et al*. 2011).

The oscillatory electrical activity seen at the intermediate glucose concentration and its conversion into uninterrupted action potential reflects the complex cross‐talk between Ca^2+^ entry, intracellular Ca^2+^ ATPase activity and Ca^2+^‐induced (via changes in the ATP/ADP ratio) of K_ATP_ channel activity (Kanno *et al*. 2002).

Like glucose, SUs promptly and reversibly depolarize the β‐cell and initiate action potential firing in β‐cells (Fig. 1*B*), which accounts for their insulin‐releasing capacity. When applied at intermediate glucose concentrations (10 mm), they also convert oscillatory electrical activity into continuous action potential firing (Henquin, 1988; Kanno *et al*. 2002), in keeping with the idea that electrical activity transiently reactivates K_ATP_ channels until the intracellular ATP/ADP ratio has been restored (Tarasov *et al*. 2012). Conversely, addition of the K_ATP_ channel activator diazoxide suppresses glucose‐induced electrical activity in β‐cells (Henquin & Meissner, 1982). Two important conclusions can be drawn from these observations: First, K_ATP_ channel closure is involved in both the initiation and modulation of β‐cell electrical activity. Second, there must be sufficient depolarizing background in β‐cells even at low glucose to account for the rapid initiation of electrical activity upon addition of SUs.

Following its release into the bloodstream, secreted insulin acts on the target organs (chiefly the liver) where it promotes glucose storage until normal blood glucose is restored. When this has occurred, insulin secretion stops by the reversal of the process described above. Thus, insulin secretion is under feedback control via changes in plasma glucose (Fig. 1*C*).

## Variations on the K_ATP_ channel theme: the α‐cell

As mentioned above, the glucagon‐producing α‐cells are also equipped with K_ATP_ channels. At the molecular level, they are identical to those in β‐cells and they are similarly influenced by ATP and ADP (Bokvist *et al*. 1999). Unlike β‐cells, α‐cells are electrically active at low (1 mm), or even the complete absence of, glucose (Gopel *et al*. 2000). A role for the K_ATP_ channels in α‐cells may seem counterintuitive given that glucose reciprocally regulates insulin and glucagon secretion. However, studies in K_ATP_ channel knockout mice firmly establish their role in glucagon secretion. In islets from such mice, glucagon secretion is strongly inhibited at low glucose, and high glucose and tolbutamide have (unlike what is seen in control islets) little or no further glucagonostatic effect (Gromada *et al*. 2004; Munoz *et al*. 2005; Shiota *et al*. 2005; MacDonald *et al*. 2007; Cheng‐Xue *et al*. 2013). We acknowledge that glucagon secretion may also be under paracrine regulation by insulin (Unger & Orci, 2010) or somatostatin (Hauge‐Evans *et al*. 2009). Indeed, it is possible that the small inhibition that persists in K_ATP_ channel‐deficient islets (10% of that in control islets) (Cheng‐Xue *et al*. 2013) reflects such paracrine actions. However, whilst somatostatin may contribute to the glucagonostatic effect at high glucose concentrations that are associated with stimulation of somatostatin secretion, its role at low glucose (when somatostatin release is low) must be limited (Zhang *et al*. 2007; Briant *et al*. 2017; Lai *et al*. 2018; Kellard *et al*. 2020). Here we will therefore focus on the alternative possibility that the glucagonostatic actions of glucose and tolbutamide reflect direct effects within the α‐cell itself and that they are mediated by inhibition of K_ATP_ channels.

### K_ATP_ channels in α‐cells *vs*. β−cells: quantitative aspects

Glucagon secretion is traditionally measured in isolated intact pancreatic islets. Many of the earlier studies that suggest little role for the K_ATP_ channels in α‐cells were conducted on isolated cells maintained in tissue culture (Barg *et al*. 2000; Quoix *et al*. 2009), which may not be fully representative of the situation *in vivo*. When K_ATP_ channel activity is instead measured in α‐cells in acutely isolated intact islets, the resting membrane conductance was found to be 270 pS at 1 mm glucose and reduced to ∼200 pS after application of tolbutamide or increasing glucose to 6 mm (the concentration producing maximal inhibition of glucagon secretion) (Zhang *et al*. 2013). The corresponding values in β‐cells are 4 nS and 1 nS (Gopel *et al*. 1999). Thus, the net whole‐cell K_ATP_ conductance in α‐cells is only 70 pS at 1 mm glucose (∼2% of the 3 nS in β‐cells). It is likely that most of the residual K_ATP_‐independent (tolbutamide‐resistant) membrane conductance largely reflects ‘leak’ across the lipid membrane itself and around the recording electrode as it is not affected by any experimental manipulation and the giga‐seal typically has a resistance of 5–10 GΩ (i.e. 100–200 pS) (Hamill *et al*. 1981).

### Strong tonic inhibition of K_ATP_ channels in α‐cells at low glucose

The low resting conductance of α‐cells explains why they are electrically active at low glucose. Importantly, it is not simply a consequence of a low expression of the K_ATP_ channel in α‐cells. The K_ATP_ channel density can be estimated from the increase in K^+^ conductance following the wash‐out of intracellular ATP. Such experiments reveal that the K_ATP_ channel density (normalised to membrane area) is, if anything, considerably higher in α‐than in β‐cells (Bokvist *et al*. 1999), in agreement with gene expression data (DiGruccio *et al*. 2016). This suggests that the K_ATP_ channels must be under strong tonic inhibition even in α‐cells exposed to low glucose. Exactly how this occurs remains to be elucidated but it may reflect effective metabolism of fatty acids in α‐cells under hypoglycaemic conditions (Briant *et al*. 2018). Carnitine palmitoyltransferase 1 (CPT1) is a mitochondrial transmembrane enzyme responsible for the formation of acyl‐carnitine from long‐chain acyl‐coenzyme A. This enzyme is considered rate‐limiting for β‐oxidation of long‐chain fatty acids. In mice that lack *CPT1* in the α‐cells, glucagon secretion at 1 mm glucose is reduced by 40%. Thus, there is a redundancy of ATP‐producing mechanisms in α‐cells. It is therefore of interest that α‐cells (unlike β‐ and δ‐cells) also express the low‐K_m_ hexokinase‐1 (*Hk1*) in addition to glucokinase (*Gck*; a high‐K_m_ hexokinase). Genetic ablation of glucokinase in α‐cells has no effect on α‐cell electrical activity and glucagon secretion at low (1 mm) glucose but abolishes the glucagonostatic effect of high (6 and 20 mm) glucose (Basco *et al*. 2018). However, the impact of genetically ablating hexokinase‐1 in α‐cells has not yet been investigated.

**Figure 2:**
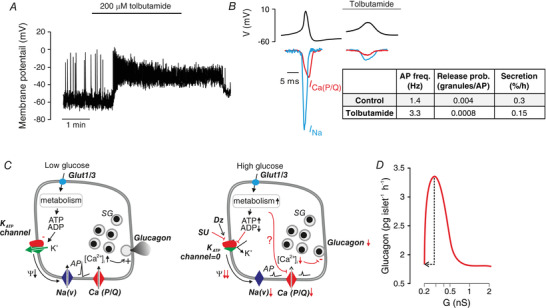
K_ATP_ channels in α‐cells *A*, effects of tolbutamide (200 μm) on action potential firing in an α‐cell within a mouse islet exposed to 1 mm glucose. Note that the α‐cell is electrically active and that tolbutamide leads to membrane depolarization and increases action potential firing. *B*, schematic of α‐cell action potentials at 1 mm glucose in the absence and presence of tolbutamide; membrane potential (*V*) shown in top trace (black) and membrane currents flowing through voltage‐gated Na^+^ channels (*I*_Na_; blue) and P/Q‐type Ca^2+^ channels (*I*_Ca(P/Q)_; red) are indicated. The table (inset, right) summarizes the action potential (AP) frequency (freq.; taken from Zhang *et al*. (2013)), release probability (prob.) and fractional glucagon release (% of content/h; estimated from reported glucagon secretion rates and islet glucagon contents (Knudsen *et al*. 2019)) and converted to number of granules released based on each α‐cell containing 7000 granules (Barg *et al*. 2000). *C*, schematic of effects of glucose and tolbutamide on electrical activity and glucagon secretion in α‐cells at low glucose (left) and at high glucose or in the presence of a high dose of SU (right). At low glucose, K_ATP_ channel activity is low but >0 and this keeps the membrane potential (Ψ) of the α‐cell sufficiently depolarized to allow AP firing whilst preventing voltage‐dependent inactivation of the voltage‐gated Na^+^ channels (Na[V]). The large‐amplitude action potentials activate the P/Q‐type Ca^2+^ channels (Ca[P/Q]). The associated increase in [Ca^2+^]_i_ triggers exocytosis of glucagon‐containing secretory granules (SGs). When glucose is elevated, increased glucose uptake via Glut1 or 3 and the associated increase in ATP/ADP_i_, K_ATP_ channel activity fall to zeros, resulting in strong membrane depolarization, inactivation of the Na(V) channels, reduced AP amplitude, less Ca^2+^ entry via Ca(P/Q) activation and a fall in [Ca^2+^]_i_, culminating in suppression of glucagon secretion. It is also possible that a glucose metabolite directly modulates Ca(P/Q) and thereby reduces Ca^2+^ entry and glucagon secretion (indicted by ‘?’). Diazoxide may reverse the glucagonostatic effect of high glucose by activation of K_ATP_ channels (black arrow) when used at an appropriate concentration leading to an increase in channel activity comparable to the decrease produced by glucose. *D*, relationship between whole‐cell K_ATP_ channel conductance (G) and glucagon secretion. The black arrow indicates changes in K_ATP_ channel activity and the resulting change in glucagon secretion when glucose is increased from 1 to 6 mm (arrow). The red curve was estimated by parallel measurements of glucagon secretion at 6 mm glucose in the presence of increasing concentrations of diazoxide (1–100 μm). Data in panel *D* from Zhang *et al*. (2013) (redrawn).

Although α‐cell K_ATP_ channel activity in the absence of glucose is very low, it is greater than zero; the observed net membrane conductance of 70 pS is equivalent to an average of five K_ATP_ channels being active in the entire cell, suggesting that the open probability of the α‐cell's >1000 K_ATP_ channels (Bokvist *et al*. 1999) is <0.5%. However, even a K_ATP_ channel activity as low as this is sufficient to keep the α‐cell membrane potential partially repolarized (∼‐55 mV) as witnessed by the 10–15 mV depolarization observed when the channels are inhibited by tolbutamide (Fig. 2*A*) or high glucose (Zhang *et al*. 2013; Babinsky *et al*. 2017). As in β‐cells, this depolarization increases action potential firing in α‐cells but this is associated with a suppression rather than stimulation of glucagon secretion. Below we discuss how this paradox arises.

### Functional consequences of K_ATP_ channel closure: role of voltage‐gated Na^+^ and Ca^2+^ channels

Glucagon secretion in α‐cells is the product of action potential frequency and the amount of glucagon secreted for each action potential. Normally, each action potential evokes glucagon granule exocytosis, albeit with a probability as low as 0.004 granules/action potential (Fig. 2*B*). Following the application of tolbutamide, the release probability is reduced by 80% (to 0.0008 granules/action potential) and although there is a 150% increase in action potential frequency, the combination of these effects produces a net 50% decrease in glucagon secretion (Zhang *et al*. 2013).

Why is the release probability reduced? The action potentials in α‐cells reflect the activation of voltage‐gated Na^+^ channels and P/Q‐type Ca^2+^ channels. Na^+^ channels (including those in α‐cells) (Zhang *et al*. 2014) have a dual dependence on membrane potential: depolarization both activates and inactivates the channels. Unlike what is observed in β‐cells where most (if not all) Na^+^ channels are locked in the inactivated state, Na^+^ channels in α‐cells exist in an ‘activatable’ state (Zhang *et al*. 2014). The α‐cells express Nav1.3 Na^+^ channels whereas Nav1.7 predominates in β‐cells but this difference is only part of the reason why the Na^+^ currents in the α‐cells inactivate at 50 mV more depolarized membrane potentials than in β‐cells (midpoint of inactivation ‐50 mV rather than ‐100 mV); most of the discrepancy is instead attributable to differences in the Na^+^ channels’ lipid environment in the α‐ and β‐cell plasma membranes (Godazgar *et al*. 2018). The central role of Na^+^ channels in the glucagon secretion at low glucose is underscored by the strong inhibition of glucagon secretion produced by the Na^+^ channel blocker tetrodotoxin (TTX) (Zhang *et al*. 2013, 2014).

Because of Na^+^ channel inactivation, the peak voltage of the action potentials declines from +10 mV when the membrane potential is ‐55 mV (or more negative) to ‐10 mV when the membrane potential is ‐45 mV (or more positive). This change in peak voltage is highly significant because exocytosis in α‐cell is steeply voltage‐dependent, largely reflecting the voltage dependence of the P/Q‐type Ca^2+^ channels that mediate the Ca^2+^ entry for glucagon secretion at low glucose (Fig. 2*B*). The change in action potential amplitude produced by application of tolbutamide can be estimated to reduce glucagon exocytosis by 80% (Zhang *et al*. 2013), similar to the estimated reduction of glucagon granules’ release probability (see above).

It is implicit from the model that exocytosis of the glucagon granules is regulated by [Ca^2+^]_i_ close to the inner mouth of the P/Q‐type Ca^2+^ channels. This is supported by the findings that exocytosis is resistant to intracellular application of millimolar concentrations of the Ca^2+^ chelator EGTA (Zhang *et al*. 2013). It remains an open question whether hyperglycaemia inhibits glucagon secretion exclusively by the same (membrane potential‐dependent) mechanism or whether glucose metabolism additionally modulates P/Q‐type Ca^2+^ channel activity by a more direct inhibitory effect. The latter possibility is suggested by studies on the glucagonostatic effects of the incretin hormone GLP‐1 (De Marinis *et al*. 2010). Such a direct modulatory effect of glucose would be consistent with the observation that glucose inhibits glucagon secretion evoked by high K^+^ (70 mm) stimulation in human islets (Ramracheya *et al*. 2018) (Fig. 2*C*), an effect that clearly cannot be accounted for by modulation of action potential height. Whether this also applies to mouse α‐cells and the underlying mechanism have not been elucidated. However, it is clear that the glucose‐induced changes in action potential height are sufficient to account for most of the glucagonostatic effect of high glucose but this does not exclude the contribution of additional mechanisms.

### Voltage‐gated K^+^ channels

Action potential repolarization in α‐cells is mediated by the activation of voltage‐gated K^+^ currents. Gene expression and electrophysiological data suggest evidence for the presence of Kv2.1/2.2 and Kv11.1/11.2 delayed rectifying and Kv4.1 (mouse) and Kv4.3 (human) A‐type K^+^ currents. Unlike what is observed in β‐cells, pharmacological inhibition of these channels in α‐cells leads to suppression of glucagon secretion at 1 mm glucose (Gromada *et al*. 2004; Ramracheya *et al*. 2010; Spigelman *et al*. 2010), possibly by resulting in a more depolarized interspike voltage that prevents complete Na^+^ channel reactivation between the action potentials (Braun & Rorsman, 2010).

### Bell‐shaped relationship between K_ATP_ channel activity and glucagon secretion: impact of pharmacological activation of K_ATP_ channels

If tolbutamide inhibits glucagon secretion, the K_ATP_ channel opener diazoxide should have the opposite effect. Indeed, when tested at *low* concentrations, diazoxide reverses the glucagonostatic effect of high glucose by reversal of the process shown in Fig. 2*C*. Notably, the concentration of diazoxide that stimulates glucagon secretion must be carefully titrated and depends on the experimental conditions such as the concentration of bovine serum albumin (commonly included in the extracellular medium in glucagon secretion studies). At high concentrations, the repolarizing effect may become so strong that any stimulatory effect on glucagon secretion due to the increase in action potential height might be cancelled out by the inhibition (partial or complete) of action potential firing. Collectively, these considerations suggest that there is a bell‐shaped relationship between K_ATP_ channel activity and glucagon secretion (Fig. 2*D*). This concept may – as we will elaborate below – have important implications for the understanding of the dysregulation of glucagon secretion associated with diabetes.

### Role of [Ca^2+^]_i_ in glucagon secretion

**Figure 3:**
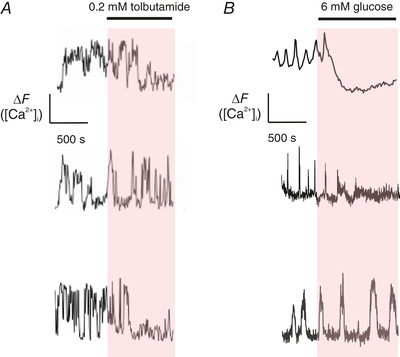
Effects of glucose and tolbutamide on [Ca^2+^]_i_ in α‐cells *A*, effects of application of 0.2 mm tolbutamide in three different α‐cells in intact islets exposed to 1 mm glucose. *B*, as in *A* but effects of increasing glucose from 1 to 6 mm. Measurements were made using fluo‐3 in *A* and the lower trace in *B*. The other measurements were performed in α‐cells expressing the Ca^2+^ sensor GCaMP3 under the proglucagon promoter.

As discussed above, changes in K_ATP_ channel activity modulate action potential firing in α‐cells. Because electrical activity involves the opening of voltage‐gated Ca^2+^ channels, electrical activity in α‐cells is associated with oscillations in [Ca^2+^]_i_ (Kellard *et al*. 2020). Despite the robust inhibitory effects of high glucose on glucagon secretion this associates with surprisingly subtle effects on [Ca^2+^]_i_ (Nadal *et al*. 1999; Quoix *et al*. 2009; Le Marchand & Piston, 2012; Zhang *et al*. 2013; Li *et al*. 2015) and although a glucose‐induced reduction of [Ca^2+^]_i_ is observed in some cells, it continues to oscillate in other cells (Fig. 3*A*). A similar variability is observed in the responses to tolbutamide when the compound is tested in intact islets (Fig. 3*B*) (Quesada *et al*. 1999). This has led to the suggestions that glucagon is regulated distal to the elevation of [Ca^2+^]_i_ by a direct effect on exocytosis (Hughes *et al*. 2018). Recently, based on imaging of several hundreds of α‐cells, we were able to demonstrate that (despite a great cell‐to‐cell variability) increasing glucose from 1 to 6 mm produced a statistically significant reduction of [Ca^2+^]_i_ manifested as a reduction of both frequency and amplitude of [Ca^2+^]_i_ oscillations (Kellard *et al*. 2020). The effect on the amplitude of the [Ca^2+^]_i_ oscillations resembles that produced by blocking the subset of Ca^2+^ channels (P/Q‐type) linked to glucagon secretion at low glucose (De Marinis *et al*. 2010), in keeping with the idea that the effect of glucose culminates in reduced Ca^2+^ entry.

In addition to the P/Q‐type Ca^2+^ channels, α‐cells are also equipped with L‐type Ca^2+^ channels. Although these channels contribute to action potential firing in α‐cells, blocking them with isradipine or nifedipine does not affect glucagon secretion at 1 mm glucose (MacDonald *et al*. 2007; De Marinis *et al*. 2010). However, L‐type Ca^2+^ channels play a key role in glucagon secretion evoked by adrenaline (and presumably other agents increasing intracellular cAMP) (De Marinis *et al*. 2010) by promoting intracellular Ca^2+^ release (Hamilton *et al*. 2018).

Consistent with a bell‐shaped relationship between K_ATP_ channel activity and α‐cell [Ca^2+^]_i_/glucagon secretion, diazoxide abolished [Ca^2+^]_i_ oscillations at low glucose (Le Marchand & Piston, 2012) but was without consistent inhibitory effect or even increased their frequency at high glucose in some cells (Li *et al*. 2015; Quesada *et al*. 1999; MacDonald *et al*. 2007), whereas complete inhibition of the β‐cells was observed. The latter difference may be related to the fact that diazoxide's capacity to activate the K_ATP_ channels is diminished by high intracellular ATP concentration (Zunkler *et al*. 1988), a concept that would be consistent with the strong tonic inhibition of K_ATP_ channels in α‐cells.

### Comparison of secretory responses in α‐ and β‐cells

Clearly, the mechanism we propose for glucose‐ and tolbutamide‐induced inhibition of glucagon secretion bears a strong resemblance to that which we outline for biphasic insulin secretion (see Abstract illustration). During sustained glucose or SU stimulation of β‐cells, there is a time‐dependent reduction in action potential amplitude as well as frequency because of voltage‐dependent and Ca^2+^‐dependent inactivation of Ca^2+^ and Na^+^ channels (partial or complete, respectively). As in α‐cells, exocytosis is β‐cells is voltage‐dependent (Gopel *et al*. 2004) and depends on the local Ca^2+^ concentration at the release sites (Barg *et al*. 2001). This will reduce the rate of insulin because of two synergistic effects: fewer Ca^2+^ channels remain to be activated (inactivation) and there is less voltage‐dependent activation of these (reduced action potential amplitude). It has been argued that this (in combination with the depletion of a pool of readily releasable secretory granules) contributes to the termination of first phase insulin secretion and that second phase secretion results from the generation of smaller action potentials (Rorsman & Ashcroft, 2018). Importantly, the rate of insulin release during the second phase remains much higher than the very low rate of release under basal conditions prior to the initiation of electrical activity. As discussed below, this is highly significant for an understanding of why glucose differentially regulates insulin and glucagon secretion.

In α‐cells, the application of tolbutamide and high glucose also leads to a reduction of action potential height. As in β‐cells, this leads to a decrease in exocytosis. However, because α‐cells (unlike β‐cells) are electrically active at low glucose, ‘basal’ glucagon secretion is high and the reduction of action potential height therefore leads to a net inhibition of glucagon secretion (as discussed above) (see Abstract illustration). In conclusion, time‐dependent changes in action potential frequency and amplitude shape the secretory responses in both β‐ and α‐cells.

## K_ATP_ channels also control glucagon secretion in humans

**Figure 4:**
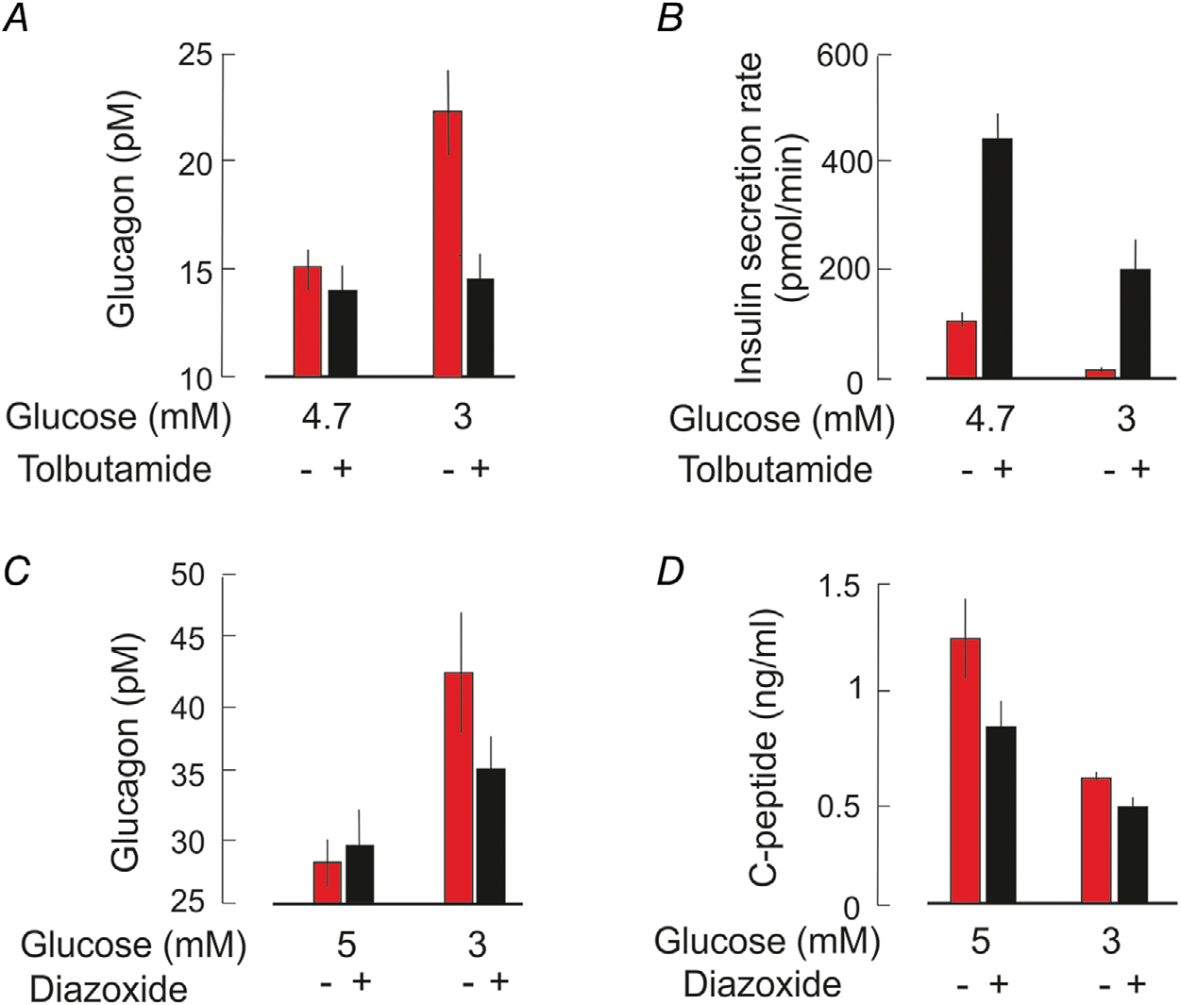
Effects of tolbutamide and diazoxide on glucagon secretion in clinical studies *A–B*, glucagon (*A*) and insulin secretion rate (*B*; estimated from the release of C‐peptide, a proxy for endogenous insulin secretion) measured in the absence and presence of tolbutamide (1 g/h intravenously) under essentially normo‐ (4.7 mm) and hypoglycaemic (3 mm) conditions. Hypoglycaemia induced by administering insulin intravenously (1.5 or 6 pmol kg^‐1^ min^‐1^). Data from Banarer *et al*. (2002) (redrawn). *C–D*, as in *A–B* but measured in the absence and presence of diazoxide (6 mg kg^−1^, orally). Data from Raju & Cryer (2005) (redrawn).

The model outlined above for the regulation of glucagon secretion is largely based on studies of α‐cells in isolated mouse islets. However, key elements of the model have been confirmed in isolated human pancreatic islets; whereas tolbutamide depolarizes human α‐cells and inhibits glucagon secretion (Ramracheya *et al*. 2010), low concentrations of diazoxide reverse the glucagonostatic effect of high glucose (MacDonald *et al*. 2007). Importantly, there are also clinical data to support the model. In healthy individuals, glucagon secretion increases when plasma glucose is lowered to ≤3.6 mm (by administration of exogenous insulin; Banarer *et al*. (2002)). Parenthetically, the fact that insulin is commonly used to produce hypoglycaemia and stimulate glucagon secretion militates against the prevailing dogma that insulin is a major glucagonostatic factor (Unger & Orci, 2010). This stimulation is abolished in the presence of tolbutamide, suggesting that counter‐regulatory glucagon secretion is mediated by an increase in K_ATP_ channel activity. Notably, tolbutamide only affects glucagon secretion at low glucose and it is ineffective under normoglycaemic conditions (Fig. 4*A*), presumably because the K_ATP_ channels are already (nearly) maximally inhibited under the latter condition. Application of tolbutamide leads to stimulation of the insulin secretion rate (estimated from the increase in plasma C‐peptide, which reflects endogenous insulin release; exogenous insulin will not contain C‐peptide); this effect was strongest under normoglycaemic conditions and in terms of absolute magnitude it was reduced by nearly 50% following induction of hypoglycaemia (Fig. 4*B*). Although the data were interpreted in terms of intra‐islet insulin exerting a glucagonostatic effect (Banarer *et al*. 2002), the findings that K_ATP_ channels are present in human α‐cells and control glucagon secretion rather suggest that it reflects a direct effect of the SU on the α‐cells. In agreement with this idea, the K_ATP_ channel activator diazoxide also inhibits glucagon secretion evoked by hypoglycaemia without affecting intra‐islet insulin secretion (again estimated from C‐peptide measurements) (Fig. 4*C–D*) (Raju & Cryer, 2005). Diazoxide reduced C‐peptide by 50% whilst not affecting glucagon secretion when applied under normoglycaemic conditions. By contrast, when the K_ATP_ channel opener was tested after induction of hypoglycaemia there was no effect on endogenous insulin release and yet counter‐regulatory glucagon secretion was reduced. The finding that both K_ATP_ channel activators and inhibitors inhibit glucagon secretion becomes understandable from the bell‐shaped relationship documented *in vitro* (Zhang *et al*. 2013).

## Islet K_ATP_ channels and diabetes: pathophysiological and therapeutic implications

There are – broadly speaking – two forms of diabetes: type 1 diabetes (T1D) that has been attributed to the autoimmune destruction of the β‐cells (resulting in a dramatically reduced insulin content) (Brissova *et al*. 2018) and type‐2 diabetes (T2D) that is caused by insufficient insulin secretion (often but not always combined with insulin resistance) (Ahlqvist *et al*. 2018). In T2D, the capacity of glucose to evoke insulin secretion is selectively reduced whereas the insulinotropic capacity of other secretagogues like GLP‐1 and arginine are maintained (Ward *et al*. 1984; Deacon & Holst, 2013). These compounds stimulate insulin secretion by mechanisms that are (partially or wholly) independent of K_ATP_ channel closure (Shigeto *et al*. 2015). This raises the interesting possibility that the impairment of glucose‐induced insulin secretion in T2D is caused by the failure of glucose to inhibit the K_ATP_ channel (possibly reflecting impaired mitochondrial ATP production) (Del Guerra *et al*. 2005). This would account for the therapeutic capacity of the K_ATP_ channel‐blocking SUs; by closing the K_ATP_ channel they restore action potential firing and insulin secretion in T2D patients. A problem with the SUs is that they, because of their mode of action (inhibiting the K_ATP_ channels by a direct/non‐metabolic effect), disable the normal feedback control of β‐cell electrical activity and insulin secretion. Consequently, insulin secretion remains inappropriately stimulated even after normal plasma glucose has been restored or even fallen below the normal range (see Fig. 4*B*).

**Figure 5:**
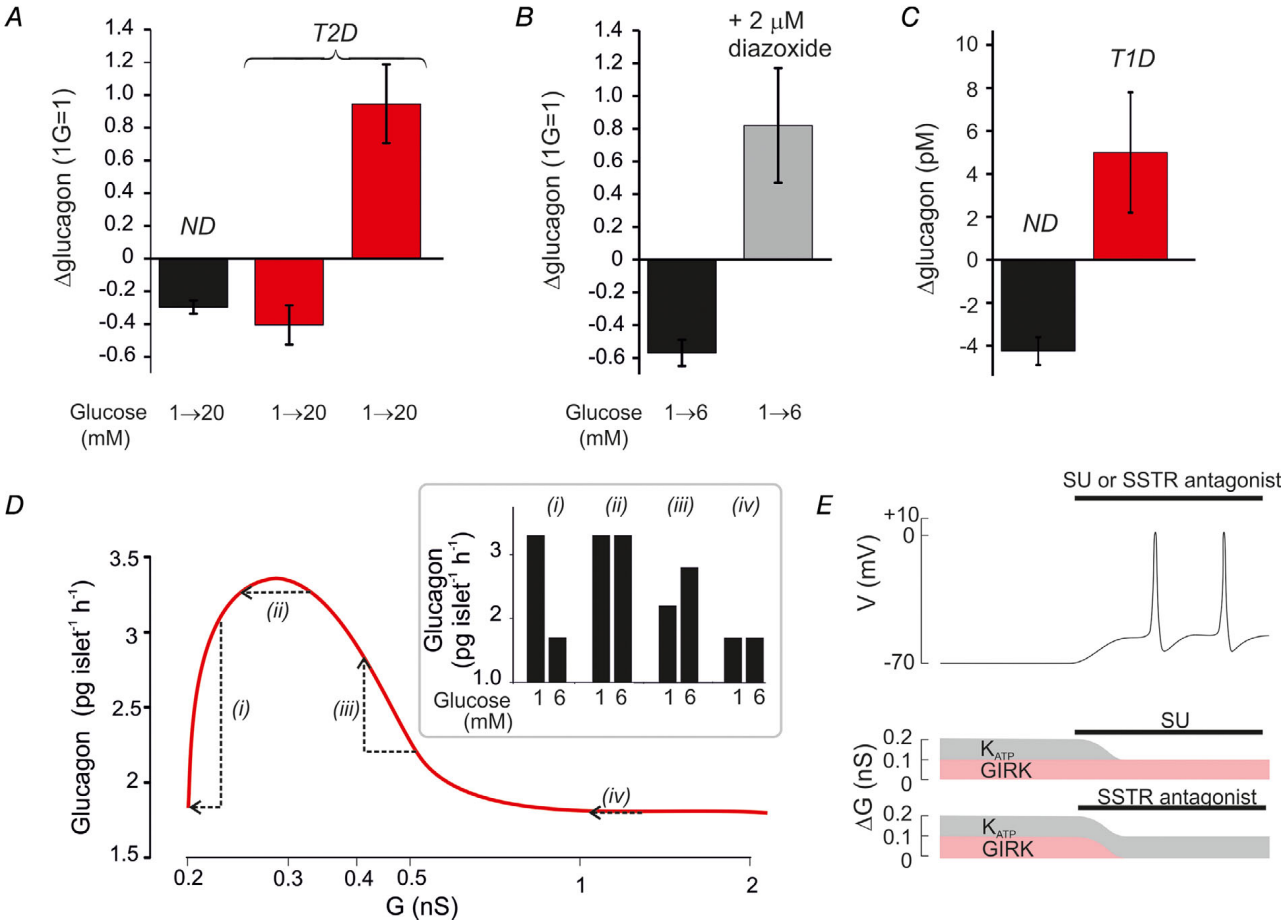
K_ATP_ channels and dysregulation of glucagon secretion in diabetes *A*, relative change in glucagon secretion measured in islets from healthy donors (*n* = 40) and donors diagnosed with T2D (*n* = 10) in response to an elevation of glucose from 1 to 20 mm. Responses in T2D islets fell into two groups; one with normal regulation (*n* = 5) and one with inverted regulation (*n* = 5). *B*, glucagon secretion in islets from two healthy organ donors at 1 and 6 mm glucose in the absence and presence of 2 μm diazoxide. *C*, net change of plasma glucagon measured in healthy individuals and in patients with T1D 4 h after administration of the SU glimepiride (4 mg, orally). Data from Cooperberg & Cryer (2009) (redrawn). *D*, relationship between total whole‐cell K channel conductance (G) and glucagon secretion (continuous curve) in T2D patients with normal K_ATP_ channel regulation (*i*) and with small (*ii*), moderate (*iii*) or large (*iv*) increases in K_ATP_ channel activity. The arrows indicate the changes in glucagon secretion produced by an elevation of glucose from 1 to 6 mm. The bar graphs (Inset) show the predicted effects of increasing glucose with variable degrees of basal K_ATP_ channel hyperactivity. *E*, schematic illustrating the effects of SU or an SSTR antagonist. The α‐cell is initially repolarized (top trace) because resting whole‐cell K^+^ conductance in excess of background (ΔG) is increased from the optimal 0.1 nS to 0.2 nS (see Fig. 2). This increase may result from either an increase in K_ATP_ (grey; due to a metabolic defect in the α‐cells) and/or GIRK (red; due to increased somatostatin release from δ‐cells) channel activity. Inhibition of K_ATP_ channels with SU (middle) or GIRK channels with an SSTR antagonist reduces ΔG to the 0.1 nS, which is optimal for α‐cell electrical activity and glucagon secretion.

Although it has attracted much less attention, both T1D and T2D are also associated with defective glucagon secretion. The dysregulation is dual: too much glucagon is secreted under hyperglycaemic conditions (when it is not needed) and too little is released in response to hypoglycaemia (when it is needed) (Muller *et al*. 1970). A similar dysregulation is seen in 50% of human islet preparations from donors diagnosed with T2D (Zhang *et al*. 2013): in these preparations glucagon secretion at 1 mm glucose is lower than in islets from non‐diabetic donors and increasing glucose stimulates rather than inhibits glucagon release (Fig. 5*A*). These defects can be recapitulated in islets from non‐diabetic donors by exposure to low concentrations of diazoxide (Fig. 5*B*) or by metabolical poisoning (Zhang *et al*. 2013), suggesting that the glucagon secretion defects in T2D result from an increase in K_ATP_ channel activity due to metabolic derangement in the α‐cell interfering with ATP production (Knudsen *et al*. 2019). In patients with T1D, glucagon secretion is completely refractory to changes in plasma glucose (Gerich *et al*. 1973).This may be also due to an increase in α‐cell K_ATP_ channel activity. This would explain the observation that a high dose of the SU glimepiride stimulates glucagon secretion in T1D patients whereas it slightly inhibits the release of the hormone in healthy individuals (Cooperberg & Cryer, 2009) (Fig. 5*C*). We attribute the weak effect of glimepiride in the healthy controls to the fact that this experiment was performed under normoglycaemic conditions (5 mm).

Based on the bell‐shaped relationship between K_ATP_ channel activity and glucagon secretion, we propose that glucagon secretion is suppressed under hypoglycaemic conditions in diabetic patients because K_ATP_ channel activity has increased beyond the optimal range (Fig. 5*D*). The severity of the metabolic defect will determine the clinical phenotype and range (depending on the increase in K_ATP_ channel activity) between nearly normal (*i*), high rate of glucagon secretion but no effect of glucose (*ii*), inverted glucose responses (*iii*) and low rate of glucagon secretion with no effect of glucose at all (*iv*) as illustrated schematically in Fig. 5*D* (inset).

These considerations suggest that it may be possible to pharmacologically restore normal glucose regulation of glucagon secretion using SUs. There is evidence from studies in isolated islets from donors diagnosed with T2D that this works *in vitro* (Zhang *et al*. 2013). It will be interesting to establish whether this is also the case in T1D islets. However, for SUs to be effective in patients with diabetes and impaired glucagon secretion, they must be carefully dosed to reduce K_ATP_ channel activity into the range where glucagon secretion is maximal; the high concentrations normally used for stimulating insulin secretion will probably inhibit glucagon secretion by producing complete inhibition of the K_ATP_ channels. In fact, such an effect may contribute to the (unacceptably) high risk of hypoglycaemia associated with SU therapy (Ruan *et al*. 2020), which is one of the reasons these compounds are no longer favoured in T2D therapy (Khunti *et al*. 2018). This is because not only do they stimulate the release of insulin under hypoglycaemic conditions (see above), they also abolish the body's counter‐regulatory response.

We point out that the bell‐shaped relationship between K^+^ channel activity and glucagon secretion is agnostic with regard to the type of K^+^ channels involved. We have previously proposed that the resting K^+^ conductance in α‐cells may be a mosaic of different K^+^ channel activities (Briant *et al*. 2016). In healthy α‐cells, K_ATP_ channels predominate but the situation might be different after the onset of diabetes (Fig. 5*E*). Indeed, T2D is associated with hypersecretion of somatostatin at low glucose (Vergari *et al*. 2020). Somatostatin inhibits glucagon secretion by several mechanisms including the activation of G protein‐coupled inward‐rectifying K^+^ channels (GIRK) and a direct effect on exocytosis (Rorsman & Huising, 2018). Interestingly, the somatostatin receptor (SSTR) antagonist CYN154806 restores glucagon secretion at 1 mm glucose in diabetic mice and in some islet preparations from donors with T2D (Vergari *et al*. 2020). In such patients, blocking the K_ATP_ channel component, low‐dose SUs might lower the resting K^+^ conductance sufficiently to stimulate secretion at 1 mm glucose but it would not correct the suppression due to inhibition of exocytosis. This would account for the fact that SUs, whilst restoring normal glucose regulation, fail to normalise glucagon secretion at 1 mm glucose to the level seen in islets from healthy mice/organ donors without T2D (Knudsen *et al*. 2019). Conversely, an SSTR antagonist would prevent activation of the GIRK channels and reverse the effect on exocytosis but not the metabolic defect leading to increased K_ATP_ channel activity. Thus, a combination of SSTR antagonists and SUs may be required to fully restore normal metabolic regulation of glucagon secretion in patients with T1D and T2D.

## Yin and Yang of K^+^ channel activity

In this Topical Review we show – based on observations in pancreatic α‐ and β‐cells – that K^+^ channel closure has a dual effect on electrical excitability and hormone/neurotransmitter release. Normally, a reduction of K^+^ channel activity increases electrical excitability by producing membrane depolarization and increasing the membrane resistance (as exemplified by the β‐cell). However, K^+^ channel activity must not be reduced too much; excessive and maintained membrane depolarization may lead to voltage‐dependent inactivation of the membrane conductances involved in action potential firing, resulting in a paradoxical decrease in electrical excitability (as exemplified by the α‐cell). Under these conditions, (high membrane) resistance is indeed futile. Thus, there is a yin and yang of K^+^ channel activity and electrical excitability. Although our argument here focuses on the role of K_ATP_ channels and the pancreatic islet α‐ and β‐cells, the concept might be equally applicable to other types of K^+^ channel and other cell types/tissues. For example, in the brain's supra‐chiasmatic nucleus, closure of small conductance Ca^2+^‐activated K^+^‐channels causes membrane depolarization and yet suppresses action potential firing (Belle *et al*. 2009). Our data suggest that the glucagon‐secreting α‐cells are regulated similarly and do not obey conventional electrophysiological expectations.

## The way forward

We acknowledge that this Topical Review has a slant towards electrophysiological explanations. However, this does not mean that events beneath the plasma membrane are irrelevant or uninteresting! As we have tried to discuss above, there must be metabolic differences between the β‐ and α‐cells that ensure that ATP is maintained at sufficiently high levels to keep the K_ATP_ channels almost fully closed even during protracted hypoglycaemia to ensure continuous release of glucagon but how this occurs is not known. If this mechanism becomes defective in diabetes, it might explain the dysregulation of glucagon secretion associated with the disease and the loss of appropriate counter‐regulatory glucagon secretion in some of the patients.

It is currently unknown whether the glucagon secretion defects associated with diabetes develop in α‐cells independently of the disruption of β‐cell function or whether they are a consequence of diabetes. Data from mouse models of diabetes suggest that the dysregulation of glucagon secretion is secondary to hyperglycaemia‐induced intracellular acidification of the α‐cell that interferes with mitochondrial ATP production that in turn results in K_ATP_ channel activation and suppression of glucagon secretion (Knudsen *et al*. 2019). It will be important to establish whether this also applies to human α‐cells and whether these defects can be reversed by pharmacological interventions. The finding that low concentrations of SUs partially correct the glucagon secretion in diabetic islets *in vitro* suggests that this is feasible (Zhang *et al*. 2013) but whether these findings can be translated into improved clinical management remains to be established in clinical trials. Importantly, the SUs must be carefully dosed to reduce K_ATP_ channel activity by ∼50% (IC_50_) as higher concentrations inhibit glucagon secretion (Zhang *et al*. 2013). A stable plasma concentration around the IC_50_ is difficult to achieve therapeutically with currently available SUs and small changes in the concentration will have large effects on K_ATP_ channel activity/glucagon secretion. Successful translation into the clinic may therefore require a K_ATP_ channel blocker with a mode of action distinct from that of the currently used lipophilic SUs that reach the binding site following solvation in the plasma membrane (Zunkler *et al*. 1989).

Finally, impaired counter‐regulatory glucagon secretion with the risk of severe (potentially fatal) hypoglycaemia represents a barrier towards optimal glycaemic control. The only effective treatment available for recurrent life‐threatening hypoglycaemia is islet or pancreas transplantation but the need for lifelong immunosuppression makes this a last resort (Harlan, 2016). Importantly, not all patients with diabetes experience severe hypoglycaemia and 80% of patients with T2D and 60% of patients with T1D seem protected even after 5 years of insulin therapy (UK Hypoglycaemia Study(Group, 2007). Why hypoglycaemia only affects some patients is not known but it is tempting to speculate that this subgroup is genetically predisposed. Whilst there has been much progress in the understanding of the genetic basis of the insulin secretion defects in T2D (Mahajan *et al*. 2018), hardly anything is known about the genetics of the glucagon secretion defects. With the advent of human iPSC‐derived glucose‐responsive α‐cells with electrophysiological properties that closely resemble those of primary human α‐cells (Peterson *et al*. 2020), it might be possible to compare glucagon secretion in response to hypoglycaemia and its relationship to electrical activity in α‐cells derived from diabetes patients with good glycaemic control and those with an elevated risk of hypoglycaemia. This represents an exciting – but challenging – area of future research into the dysregulation of glucagon secretion in diabetes.

## Additional information

### Competing interests

The authors declare no competing interests.

### Author contributions

QZ and HD researched the data. PR drafted the manuscript, which was edited and approved by all authors.

### Funding

Supported by Diabetes UK (QZ), the Swedish Research Council (PR) and the Helmsley Trust (PR). Concepts presented in this Topical Review are based on studies supported by the Wellcome Trust.
